# Using Visual Feedback Manipulation in Virtual Reality to Influence Pain‐Free Range of Motion in People with Nonspecific Neck Pain

**DOI:** 10.1111/papr.12971

**Published:** 2020-12-20

**Authors:** Maaike Kragting, Stefan F. Schuiling, Lennard Voogt, Annelies L. Pool‐Goudzwaard, Michel W. Coppieters

**Affiliations:** ^1^ Department of Physical Therapy Research Centre for Health Care Innovations Rotterdam University of Applied Sciences Rotterdam The Netherlands; ^2^ Department of Human Movement Sciences Faculty of Behavioural and Movement Sciences Amsterdam Movement Sciences Vrije Universiteit Amsterdam Amsterdam The Netherlands; ^3^ Pain in Motion Research Group Department of Physiotherapy, Human Physiology and Anatomy Faculty of Physical Education & Physiotherapy Vrije Universiteit Brussel Brussels Belgium; ^4^ Menzies Health Institute Queensland Griffith University Brisbane and Gold Coast Queensland Australia

**Keywords:** classical conditioning, illusion, visual feedback manipulation, extended reality (XR), rehabilitation, disability

## Abstract

**Background:**

Based on associative learning theories it is hypothesized that pain might be a conditioned response. In people with musculoskeletal pain, the occurrence of movement‐induced pain might be a protective response, influenced by visual cues suggesting that the person is approaching a painful position. This study aimed to determine (1) whether the pain‐free range of motion (ROM) increased and decreased when visual feedback understated or overstated true rotation in people with neck pain and (2) whether this effect was more pronounced if pain was chronic.

**Method:**

People with subacute and chronic nonspecific neck pain wore a VR‐headset and rotated their head to the left and right until the onset of pain. Visual feedback about the amount of movement was either equal, 20% less, or 20% greater than their actual rotation. Maximal pain‐free ROM was measured using the VR‐headset sensors. Data were analyzed using a mixed‐design ANOVA.

**Results:**

There was no effect of visual feedback manipulation on pain‐free ROM (*P* = 0.13) and no interaction effect between the visual feedback condition and duration of pain (*P* = 0.86).

**Discussion:**

The inability to influence pain‐free ROM by manipulating visual feedback in people with subacute or chronic neck pain does not support associative learning theories for the perception of neck pain.

## Introduction

Musculoskeletal pain is a complex phenomenon of which the underlying mechanisms are still not fully understood.^1^, ^2^ Although it is well established that psychological factors^1^, ^2^, ^3^, ^4^ and contextual factors^5^, ^6^, ^7^, ^8^ play an important role in musculoskeletal pain, it is still a quest how exactly these factors contribute to the perception of pain.^9^, ^10^, ^11^

Classical conditioning has been proposed as a relevant theory that explains how psychological learning processes contribute to the perception of musculoskeletal pain.^12^, ^13^ Based on this theory, it is hypothesized that proprioceptive signals (eg, movement and posture) and exteroceptive signals (eg, visual, tactile, auditory), which are accompanied by a nociceptive signal, can be associated with the perception of pain.^12^, ^13^ According to this hypothesis, multisensory and meaningful events that coincide with nociceptive information are considered the conditioned stimulus, while pain is considered to be the conditioned response. This hypothesis proposes that pain perception is, at least partially, influenced by associative learning mechanisms, and various experiments using experimental pain support this hypothesis.^10^, ^14^, ^15^

In line with this hypothesis, there is preliminary evidence from a single trial involving people with nonspecific neck pain that the position at which pain occurs during movement might be a conditioned response.^16^ This study showed that modifying visual feedback during movement in a virtual reality (VR) environment (ie, altering a non‐nociceptive stimulus) influences the pain‐free range of motion. When visual feedback overstated true rotation, the pain‐free range of motion decreased by 7% (or a mean absolute decrease of 2.6° in left rotation and 4.3° in right rotation), while when visual feedback understated true rotation, the pain‐free range of motion increased by 6% (or a mean absolute increase of 3.3° in left rotation and 1.6° in right rotation) (Ref. 17 and personal communication). This suggests that the moment when pain is first felt while rotating the head is influenced by the visual perception of the amount of rotation. These results are encouraging because they provide insight into the role of visual information and the underlying mechanisms in pain perception, which may have implications for treatment. However, the sample size was small (*N* = 24), and the magnitude and even the direction of the effect varied considerably between participants.^16^, ^17^ Therefore, more research is required to test the hypothesis that altered visual feedback can influence the pain‐free range of motion in people with nonspecific neck pain.

The current study was conducted to verify the effect of altered visual feedback on the pain‐free range of motion in a larger group of people with nonspecific neck pain and to determine whether the duration of symptoms had an influence on the effect. We hypothesized that the magnitude of the effect of altered visual feedback would be larger in people with chronic pain than in people with subacute pain. This hypothesis is based on the assumption that in people with persistent pain associative learning might have become more entrenched.^11^, ^18^ In chronic pain, pain perception is more dissociated from noxious stimuli and the influence of other (non‐nociceptive) stimuli may become more important.^19^ In subacute pain, the noxious stimulus may still be present and associated with pain.

Therefore, the primary aim of this study was to determine whether the pain‐free range of motion increased and decreased when visual feedback understated or overstated true neck rotation. The secondary aim was to explore whether people with long‐lasting chronic neck pain were more prone to the effect of visual feedback manipulation than people with subacute neck pain. The tertiary aim was to monitor motion sickness because this is a common side effect in the use of VR,^20^ especially in people with neck pain,^21^ and an important factor when considering implementing VR in clinical practice.

## Methods

A cross‐sectional, multicenter, experimental trial was conducted in The Netherlands to address the aims of the study. The protocol of this study was approved by the Scientific and Ethical Review Board (VCWE) of the Faculty of Behavioural and Movement Sciences, Vrije Universiteit Amsterdam, The Netherlands (VCWE‐2016‐218R1).

### Participants

Participants were recruited from six primary‐care physiotherapy clinics in Rotterdam and surroundings. The treating physiotherapist performed the initial screening. Inclusion criteria were as follows: (1) neck pain Grade I (ie, neck pain with no signs of major pathology and no or little interference with daily activities) or Grade II (ie, neck pain with no signs of major pathology but with interference with daily activities)^22^ that was provoked and/or aggravated by cervical rotation, (2) aged between 18 and 65, and (3) being able to read and understand Dutch. Participants were excluded if they had neck pain with neurological signs (ie, neck pain Grade III) or neck pain with signs of serious pathology (ie, neck pain Grade IV).^22^ People with impaired vision (eg, people who had poor vision when not wearing their glasses) were also excluded. When meeting the selection criteria, participants received an information letter containing all relevant information, without details about the aims of the study and exact procedures to ensure blinding of participants. Written informed consent was obtained prior to study participation.

### Demographics and Questionnaires

All participants completed a digital questionnaire in the week prior to the experiment using an online survey system (Qualtrics, Provo, UT, U.S.A.). Information from this questionnaire was used to describe the characteristics of the participants and contained the Dutch Version of the Neck Disability Index (NDI‐DV),^23^ a Numeric Pain Rating Scale (NPRS), the duration and onset of their neck pain (gradual or sudden, and if sudden, history of trauma), and demographic questions regarding age and sex. The NDI‐DV is a reliable^24^ and widely used tool to assess the level of disability in people with neck pain. Identical to the English‐language version, it consists of 10 items with six response categories (range 0 to 5, total score range 0 to 50, with higher scores representing higher disability^24^). The NPRS is a simple and valid tool to measure pain intensity on an 11‐point scale,^25^ which has slightly superior measurement properties as compared with other pain scales.^26^

The duration of the neck pain was used to create three subgroups, ie, people with subacute neck pain (3 weeks to 3 months), chronic neck pain (3 to 24 months), and long‐lasting chronic neck pain (> 24 months).

At the completion of the experiment, participants completed the Short version MIsery SCale (sMISC) (Refs. 27, 28 and personal communication) to explore whether VR induced motion sickness or nausea. The sMISC assesses feelings of misery, is easy to administer, and correlates strongly with the more extensively validated Simulator Sickness Questionnaire.^27^, ^29^ The sMISC is scored on a six‐point scale in which 0 = no symptoms, 1 = mild symptoms (eg, dizziness, blurred vision, sweating, feeling cold, fatigue), but no nausea, 2 = severe symptoms, but no nausea, 3 = mild nausea, 4 = severe nausea and 5 = vomiting.

### Protocol

**Figure 1:**
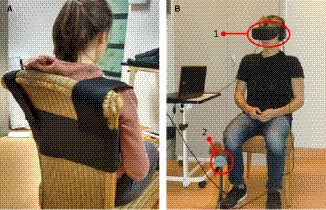
(A) The participant sat on a chair and wore a fixation belt over the shoulders and upper torso to prevent trunk rotation. (B) The participant wore the VR‐headset (1) and sat in front of the Oculus‐sensor (2).

**Figure 2:**
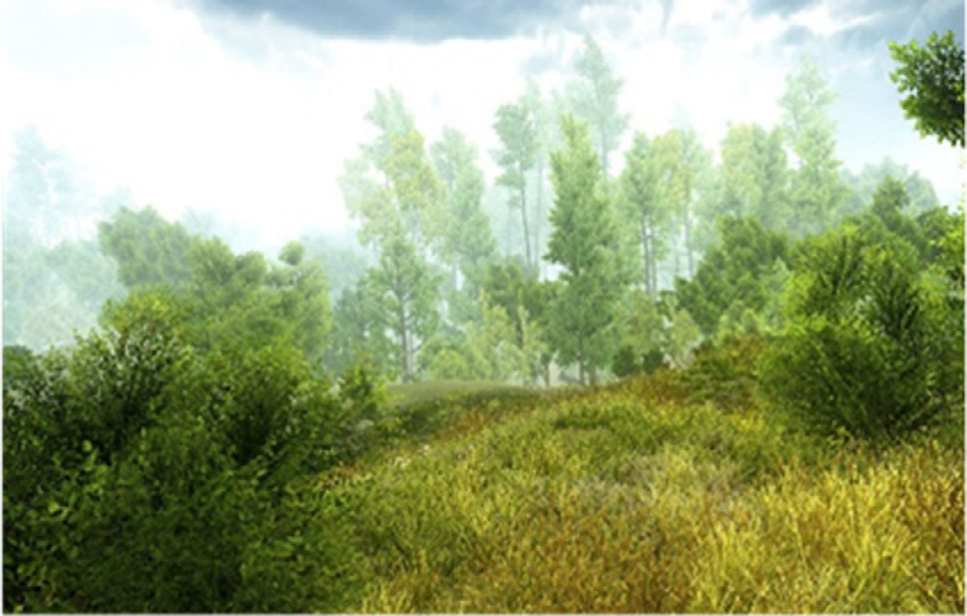
Example of the virtual reality environment projected in the VR‐headset.

Participants were tested in the physiotherapy clinic where they were recruited. Participants sat on a chair with their upper trunk fixated to limit movements of their torso (see Figure 1). They wore a VR‐headset (Oculus Rift head‐mounted display; Oculus VR, Irvine, CA, U.S.A.), which was connected to a computer running Windows. Participants were submerged in a virtual forest (see Figure 2),^30^ using Unity 5.3.1 software (Unity Technologies, San Francisco, CA, U.S.A.). This virtual environment was selected because this scenery had no obvious reference points that could be easily remembered. Participants wore noise cancelling headphones to reduce ambient noise. Soft and consistent nature music was played via the headphones during the experiment. The music was held constant between the conditions. Prerecorded audio files with instructions were used to improve standardization.

Three conditions were tested, in which the visual feedback about the amount of movement was either less, equal, or greater than the actual physical rotation. For the rotation gains (ie, the factor that transforms actual neck rotation to the visual rotation as shown by the VR‐headset (Gain_rot_ = Rot_virtual_/Rot_real_)),^16^ a VR technique named redirected walking^31^ was used. The three gain settings were: gain of 1.0 (ie, undistorted visual feedback), gain of 0.8 (ie, 20% less visual rotation feedback than actual rotation), and gain of 1.2 (ie, 20% more visual rotation feedback than actual rotation). Manipulations with gains of 0.8 and 1.2 are typically unnoticeable by participants.^16^

Participants faced forward and were asked to rotate their head slowly to the left until the onset of pain, then to the right until the onset of pain, and then back to the midline. The total range of motion (ie, from maximal left rotation to right rotation) was calculated. The gain was changed every two repetitions, following a fixed sequence: 0.8 gain, 1.0 gain, 1.2 gain. Each participant performed 18 repetitions (six repetitions per gain condition).

After every second repetition, the participants were placed in a different location in the virtual forest to prevent them from remembering their previous end of rotation point. Three different locations in the forest were used. The locations were selected in a semirandom order, but an environment could not be selected more than twice consecutively. There was a 2‐minute rest period after six repetitions to minimize motion sickness and to score the pain intensity during the experiment. During this interval, participants kept the VR‐headset on and had their eyes closed.

The participants were blinded to the gain conditions. The researchers were not blinded for the order of the conditions, but were unable to influence the results because of the standardized prerecorded instructions delivered via the headphones and the automated recording of the range of rotation via the sensors in the VR‐headset (see below). Data per participant were extracted after the completion of the experiments at one physiotherapy clinic.

### Data Acquisition and Analysis

#### Range of Motion

Maximal pain‐free range of rotation was measured in degrees using the sensors in the VR‐headset. A pilot study was conducted to verify whether the rotation measured by the sensors in the Oculus Rift headset was valid. Therefore, two active markers of the Optotrak 3020 computerized tracking system (Northern Digital, Waterloo, ON, Canada) were placed symmetrically on the top of the VR‐headset. Then, 20 rotations were performed, and the amplitudes measured by the VR‐headset were compared with those measured by the Optotrak system. A Pearson correlation coefficient was used to determine the linear dependence between the two measurement systems (the data were normally distributed). This resulted in a perfect correlation coefficient of 1.00, *P* < 0.001. Please see Appendix S1 for further details.

In the main study, data from the VR‐headset were extracted off‐line using a custom‐written Matlab program to calculate the range of rotation (Version R2016b; The Mathworks Inc., Natick, MA, U.S.A.). For each gain condition, the mean range of motion of six repetitions was calculated, and used for further statistical analyses.

### Statistical Analyses

To determine the effect of visual feedback manipulation on the pain‐free range of motion and to examine whether this effect was different between people with subacute neck pain (≤ 3 months), chronic neck pain (3 to 24 months), and long‐lasting chronic neck pain (> 24 months), a General Linear Model (GLM), repeated‐measures mixed‐design ANOVA was performed.^32^ Partial η^2^ was calculated to determine the effect size, and Cohen’s guidelines were used to interpret the effect size. An effect size between 0.01 and 0.059 was considered small, between 0.059 and 0.138 was considered medium, and ≥ 0.138 was considered large.^33^

The assumption of normality was assessed by visual inspection of the histograms, skewness, kurtosis, and a Kolmogorov–Smirnov test. The assumption of equality of variances between the three subgroups (subacute, chronic_≤ 24 months_, chronic_> 24 months_) on the pain‐free range of motion in the three gain conditions was checked using Levene’s test for homogeneity of variance. The assumption of sphericity was checked according to Girden.^34^ When appropriate, the mixed‐design ANOVA was followed up with simple contrasts to identify whether specific differences occurred between the three gain conditions and *r* was calculated to determine the effect size. Cohen’s guidelines (1988, 1992) were used to interpret the effect size. An effect size that varies around 0.1 was considered small, around 0.3 was medium, and around 0.5 was large.^33^, ^35^

Pain ratings were compared among each set of six repetitions, using a repeated‐measures ANOVA or, in case of violations of the normality assumptions, a Friedman’s ANOVA. For the sMISC scores, frequencies and percentages were reported because of the ordinal measurement level of this variable. All statistical analyses were conducted using SPSS (IBM Corp. Release 2016. IBM SPSS Statistics for Windows, version 23.0. Armonk, NY, U.S.A.).

The sample size was calculated a priori using an ANOVA repeated‐measures within–between interaction design in G‐Power 3.1.^36^ Because of the mixed design that was used in this study, we expected a smaller effect than the (within) effect of altered visual feedback on the pain‐free range of motion as revealed in a previous study ($\eta_{p}^{2}$ = 0.29).^16^ Based on an expected effect size of $\eta_{p}^{2}$ = 0.145 (ie, 0.29/2), a significance level of α < 0.05, a power of 1 − β = 0.8, three groups, three measurements, and assuming a 70% correlation among repeated measures, the minimum required number of participants was 60, ie, 20 per group.

## Results

Seventy‐one volunteers with nonspecific neck pain participated (50 females; mean [standard deviation, SD] age: 46.0 [10.6] years; median [interquartile range, IQR] duration of neck pain: 13.5 [57.0] months). One additional participant was unable to complete the experiment due to nausea and was excluded from the study. Regarding the history of the neck pain, two‐thirds of the participants experienced a gradual onset (63%) and one‐third a sudden onset of their neck pain (37%), mostly due to a mechanical trauma (motor vehicle accident or fall) in history (30%). Most people were moderately disabled (median NDI score = 16.0 [32%]), and the mean (SD) neck pain intensity was 5.6 (1.9; see Table 1). Twenty participants had subacute neck pain (3 weeks to 3 months), 21 participants had chronic neck pain (3 to 24 months), and 29 participants had long‐lasting chronic neck pain (> 24 months). For one participant, data regarding the duration of the neck pain were missing. Therefore, these data were excluded from the subgroup analyses.

**Table 1:** Participant Characteristics VR Gain

Variables	Total *N* = 71*	Subacute *N* = 20	Chronic_≤ 24 months_ *N* = 21	Chronic_> 24 months_ *N* = 29	Differences Between Subgroups Based on Duration of Neck Pain	
					Significance	Effect Size (*r*)
Women (*N* [%])	50 (70%)	15 (75%)	12 (57%)	23 (79%)		
Men (*N* [%])	21 (30%)	5 (25%)	9 (43%)	6 (21%)		
Gradual onset	45 (63%)	12 (60%)	9 (43%)	23 (79%)		
Sudden onset	26 (37%)	8 (40%)	12 (57%)	6 (21%)		
Trauma (car accident or fall)	21 (30%)	3 (15%)	10 (48%)	8 (28%)		
Age (in years; mean [SD])	46.0 (10.6)	45.9 (8.7)	43.7 (12.5)	47.9 (10.5)	*P* = 0.400	0.164
Duration of neck pain (in months; Mdn [IQR])	13.5 (57.0)	2.0 (2.0)	6.0 (11.0)	72.0 (228.0)		
Pain intensity (NPRS; mean [SD])						
Average last week	5.6 (1.9)	5.7 (1.7)	5.8 (1.8)	5.3 (2.2)	*P* = 0.681	0.107
Maximum last week	6.9 (1.9)	7.4 (1.3)	7.1 (1.7)	6.4 (2.3)	*P* = 0.119	0.235
Disability (NDI; Mdn [IQR]) Total score (%)	16.0 (14) (32%)	9.5 (9) (19%)	16.0 (12) (32%)	18.0 (17) (36%)	*P* = 0.032	0.312

IQR, interquartile range; Mdn, median; *N*, number; NDI, neck disability index; NPRS, numeric pain rating scale; SD, standard deviation; Sign. diff., significant difference.

* For one participant, data regarding the duration of the neck pain were missing. Therefore, this participant was not included in any of the subgroups.

Analyses showed that the three groups of participants were comparable regarding age (*F*_(2,67)_ = 0.93, *P* = 0.400) and NPRS scores (NPRS_average_: *F*_(2,67)_ = 0.39, *P* = 0.681; NPRS_maximum_: *F*_(2,64.09)_ = 2.20, *P* = 0.119) but not NDI scores (ie, disability; *F*_(2,67)_ = 3.62, *P* = 0.032, *r* = 0.312). The long‐lasting chronic_> 24 months_ neck pain group was more disabled than the subacute and chronic_≤ 24 months_ neck pain group (*P* = 0.031). There was no difference in disability between the subacute and the chronic_≤ 24 months_ group (*P* = 0.198; see Table 1).

### Statistical Assumptions

The Kolmogorov–Smirnov test showed that the distribution of the range of motion scores was not significantly different from a normal distribution (all *P* ≥ 0.057), except for the 1.2 gain condition in the subacute neck pain group (*D*(20) = 0.20, *P* = 0.03). Because the absolute values of *Z*_skew_ = −2.98 and *Z*_kurt_ = 2.07 were close to 2 and due to the lack of a nonparametric variant for the mixed‐design ANOVA, data were considered to be normally distributed. Levene’s test revealed that there were no violations of the assumption of homogeneity of variance between the gain conditions. The Greenhouse‐Geisser EPSILON was ≥ 0.75; therefore, the Huynh‐Feldt correction was used.^37^

### Effect of Visual Feedback Manipulation and Duration of Neck Pain on Pain‐Free Range of Motion

**Figure 3:**
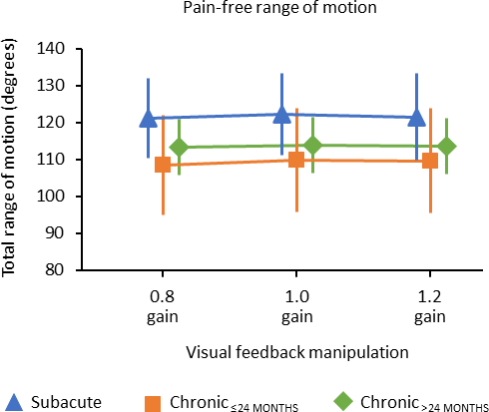
Effect of visual feedback manipulation on the pain‐free range of motion in people with subacute, chronic_≤ 24 months_, and chronic_> 24 months_ neck pain. The total range of motion is the sum of left and right rotation. The error bars represent the 95% confidence intervals.

Mean total range of motion (ie, from maximal left rotation to right rotation) was 114.1° in the 0.8 gain condition (95% confidence interval [CI]: 108.3 to 119.9), 115.0° in the 1.0 gain condition (95% CI: 109.1 to 120.9), and 114.6° in the 1.2 gain condition (95% CI: 108.6 to 120.7) (see Table 2, Figure 3 and Appendix S2: Figure S1). The ANOVA revealed that there was no significant main effect of gain on pain‐free neck range of motion *F*_(1.66,111.35)_ = 2.13, *P* = 0.133, ղ^2^ = 0.031 and no significant difference in the pain‐free range of motion between the subacute, chronic_≤ 24 months_, and chronic_> 24 months_ neck pain group (*F*_(2,67)_ = 1.34, *P* = 0.268, ղ^2^ = 0.038). There was also no significant interaction between gain and duration of neck pain (*F*_(3.32,111.35)_ = 0.27, *P* = 0.863, ղ^2^ = 0.008). This indicates that the mean pain‐free range of motion when visual feedback understates or overstates true neck rotation was not depending on the duration of the neck pain.

**Table 2:** Influence of Visual Feedback Manipulation on the Range of Motion

Gain Condition	Absolute Range of Motion (Degrees; Mean [SD])			
	Total (*N* = 71*)	Subacute (*N* = 20)	Chronic_≤ 24 months_ (*N* = 21)	Chronic_> 24 months_ (*N* = 29)
0.8 gain	113.5 (24.5)	121.2 (23.1)	108.3 (29.7)	113.4 (19.8)
1.0 gain	114.5 (25.1)	122.2 (23.8)	109.8 (30.8)	113.9 (20.0)
1.2 gain	114.1 (25.5)	121.5 (25.1)	109.5 (31.1)	113.6 (19.9)

SD, standard deviation.

* For one participant, data regarding the duration of the neck pain were missing. Therefore, this participant was not included in any of the subgroups.

### Pain Intensity

The pain intensity changed during the experiment from NPRS 4.6 [95% CI: 4.1 to 5.1] after the 6th repetition to 4.9 [95% CI: 4.3 to 5.4] after the 12th repetition and to 5.0 [95% CI: 4.4 to 5.6] after the 18th repetition. Although the mean increase in pain intensity was small (0.4/10.0 NPRS), the increase was significant (Friedman’s ANOVA χ^2^ (2) = 11.94, *P* = 0.03). Wilcoxon tests were used to follow up this finding. It appeared that pain intensity significantly increased between the 6th (median NPRS score = 5) and the 12th repetition (median = 5), *T* = 238, *P* = 0.001, *r* = 0.268, and the 6th (median = 5) and the 18th repetition (median = 5), *T* = 905, *P* = 0.002, *r* = 0.263, but not between the 12th and the 18th repetition, *T* = 505, *P* = 0.175, *r* = 0.115.

### Motion Sickness

During the VR submersion, the participants experienced no symptoms (51%), mild symptoms but no nausea (7%), severe symptoms but no nausea (10%), mild nausea (31%), or severe nausea (1%) The data for the three subgroups are presented in Table 3.

**Table 3:** Misery Scores at the End of the Experiment

sMISC Scores	Total (*N* = 71*, %)	Subacute (*N* = 20, %)	Chronic_≤ 24 months_ (*N* = 21, %)	Chronic_> 24 months_ (*N* = 29, %)
0: No nausea or other symptoms (*N* [%])	36 (51)	8 (40)	10 (48)	18 (62)
1: Mild symptoms, but no nausea (*N* [%])	22 (31)	9 (45)	6 (29)	6 (21)
2: Severe symptoms, but no nausea (*N* [%])	5 (7)	1 (5)	3 (14)	1 (3)
3: Mild nausea (*N* [%])	7 (10)	2 (10)	1 (5)	4 (14)
4: Severe nausea (*N* [%])	1 (1)	0 (0)	1 (5)	0 (0)
5: Vomiting	0 (0)	0 (0)	0 (0)	0 (0)

sMISC, short version misery scale.

* For one participant, data regarding the duration of the neck pain were missing. Therefore, this participant was not included in any of the subgroups.

## Discussion

The current research project could not confirm that the pain‐free range of motion can be altered in people with neck pain by manipulating visual feedback regarding the amount of rotation in a VR environment. Furthermore, the duration of neck pain had no impact on the effect of visual feedback manipulation on the pain‐free range of motion.

The finding that the pain‐free range of motion in people with neck pain was not influenced by the visual perception of the amount of rotation was unexpected, as it differed from earlier preliminary findings^16^ and is not consistent with the underlying hypothesis of predictive associative learning as important mechanism in pain perception.^12^, ^13^ However, a critical reflection on this hypothesis shows that associative learning is primarily considered to be important in pain‐related constructs, such as developing fear of movement and avoidance behaviour,^11^, ^38^, ^39^ while the role of associative learning in pain perception is less clear. Most studies confirm that associative learning plays a role in enhancing pain, but it is unclear whether associative learning can elicit pain.^40^ Although several studies show that a non‐nociceptive stimulus can be perceived as painful based on classical conditioning,^10^, ^15^, ^40^ other studies show opposite results.^41^, ^42^ In addition, this experimental testing of the hypothesis has been mainly based on studies with healthy participants in whom experimental pain was induced. In these studies, there was an explicit learning phase, in which the coupling between a non‐nociceptive stimulus and experimental pain was learned. Whether these findings are transferrable to clinical situations remains unclear.

The present study on the role of non‐nociceptive stimuli in the occurrence of pain is one of the few studies conducted among people with musculoskeletal pain. In the design of the current experiment, it had been assumed that associative learning plays a role and would already have been established in the persistence of neck pain. It is uncertain whether this was a correct assumption. Future experiments among people with musculoskeletal pain, using a phase in which any associations made between non‐nociceptive stimuli and pain are unlearned, may provide clarity in the role of associative learning in the persistence of pain. However, a recent study of people with chronic neck pain, based on a comparable paradigm, used VR for a longer period (ie, 21 to 28 days) to reduce any association made between visual stimuli and pain. In that study, it was expected that VR with overstated feedback would create a situation in which visual cues that were normally followed by pain were now paired with “no‐pain” and that pain would be reduced, following the extinction principle. However, even this longer phase of deconditioning did not show that this reduced pain.^43^

Another point to consider regarding the nonoccurrence of an effect of visual feedback manipulation on the range of motion in the current study is the size of the gain. Changes in the range of motion seem to depend on the size of the gain,^44^ though Harvie et al.^16^ used the same gain (0.8, 1.0 and 1.2 gain) as in the current study with different results. Further increasing the gain might be an option, although this could further increase motion sickness. In the current study, 49% of the people experienced mild (10%) or severe (1%) nausea (ie, a diffuse sensation of unease and discomfort, often perceived as an urge to vomit), or other symptoms (38%; eg, dizziness, blurred vision, feeling irritated, fatigue, sweating). This percentage is slightly lower than found in other studies investigating the possibilities for applying VR in people with neck pain.^21^

A close comparison with the study of Harvie et al.^16^ shows that the participants included in both studies were similar with respect to age (46 vs. 45 years), sex (70% vs. 75% women), NDI (32% vs. 29%), and mean duration of the neck pain (ie, chronic; 5 years in the whole group [*N* = 71] and 12 years in the chronic_> 24 months_ group [*N* = 29] vs. 11 years in the study by Harvie et al. [*N* = 24]). Furthermore, in both studies, participants had to rotate their head to the left and right, in a VR environment, until the onset of pain. Two differences were, however, present between the two studies: (1) In the study by Harvie et al.,^16^ the range of motion to the right side was measured separately from the left. A laser pointer was used to indicate the starting position, which was meant to be kept constant. However, it seems impossible to start each rotation from exactly the same point. Small deviations from this starting point could have influenced the range of motion measured. The current project measured the total range of motion from the maximum pain‐free position in the left rotation to the maximal pain‐free position in the right rotation, eliminating potentially an error with the starting position. In our opinion, this accurate measurement method, combined with the large number of participants, resulted in credible findings. However, it should be noted that in some participants rotation in one direction was more provocative than in the other direction. As we measured total range of motion, this summation of side‐specific differences might have reduced the effect of visual feedback on the total pain‐free range of motion. (2) In the current study, participants were positioned at three different places in a single VR environment (a forest), while Harvie et al.^16^ used several VR environments (a park, a mountain, countryside, church grounds, and two indoor scenes) and the same starting point for the different repetitions in the various VR environments. It could be that differences between the two studies may relate to the choice of VR characteristics, for example because the extent of subjective immersion in the VR environment might have differed. It is possible that the use of different VR scenes has influenced the results of the previous study because this might have created greater attention to the virtual environment and therefore less vigilance about range of motion and pain. Another possibility is that striking objects were present in some of these environments (eg, a building, a table, or a window). Clear reference points are important in the storage of memories^45^, ^46^ and seem to be related to associative learning.^13^, ^47^ Finally, it should be mentioned that in the majority of replication studies no effect, or a diminished effect size is found.^48^ This is consistent with the findings in the studies discussed above.

Another finding in the current experiment was that the reported pain intensity increased slightly during the experiment. This was not anticipated, because in everyday practice, exercising within the pain‐free range of movement is frequently used in the treatment of people with nonspecific neck pain and recommended in clinical guidelines.^49^, ^50^ Although it is not expected that the increase in pain during the experiment has biased the outcome, as the gain condition changed after every two repetitions, the instruction “rotate your head … and stop at the onset of pain,” might have created an unintended focus on pain.^51^, ^52^ Although an increase in pain intensity of 0.4 points on a ten‐point scale remains well within the boundaries of a minimal important difference of 1.5 to 2.5 points,^53^, ^54^ it may be important to avoid a focus on pain or cervical movements (internal focus), when further orientating on the possible added value of VR for clinical applications.^55^ This could be done by providing a more immersive and interactive VR experience in which people have to fulfil a specific task and thereby diverting attention from pain and creating a more external focus.

To conclude, contrary to our hypothesis, we could not confirm that visual feedback manipulation regarding the amount of rotation could alter the pain‐free range of motion in people with neck pain. This result was similar for people with subacute and chronic neck pain. More research, with specific adaptations (such as increasing the gain change, investigating the influence of different VR environments, and monitoring the amount and the direction of the restriction in the range of movement within participants), is needed to test the hypothesis that pain might be a conditioned response in people with nonspecific neck pain and to further unravel the role of associative learning in the persistence of pain.

## Conflict of Interest

The authors report no conflict of interest.

## Authors’ Contributions

M.K., L.V., A.P.G., and M.C. designed the study. M.K. and S.F.S. collected, analyzed, and interpreted the data. M.K. drafted the versions of the manuscript, with detailed input from M.C., L.V., and A.P.G. All authors critically reviewed and approved the final manuscript.

## Supporting information


**Appendix S1.** Technical note on the accuracy and drift of Oculus Rift.

**Appendix S2.** A boxplot showing more detailed information regarding the effect of visual feedback manipulation on pain‐free range of motion.
**Figure S1.** Effect of visual feedback manipulation on the pain‐free range of motion in people with subacute, chronic_≤ 24 months_, and chronic_> 24 months_ neck pain.

## Data Availability

The datasets and analyses are available from the corresponding author on reasonable request.
