# Supplementary material for: Using Visual Feedback Manipulation in Virtual Reality to Influence Pain‐Free Range of Motion in People with Nonspecific Neck Pain
Source: Pain Pract. 2020 Dec 20;21(4):428–37. doi: 10.1111/papr.12971 (PMC8048536; doi:10.1111/papr.12971)
Supplement: Supplementary file 1 — Appendix S1. Technical note on the accuracy and drift of Oculus Rift. [file PAPR-21-428-s001.docx]

**Appendix S1: Technical Note on the accuracy and drift of Oculus Rift**

The ROM of the participants was measured using the Oculus Rift, which provides output on pitch, roll and yaw. For the analysis of the current study, the maximal positive yaw was added to the absolute maximal negative yaw, resulting in the total cervical range of motion for sideways rotation. To make sure the yaw measured by the sensors integrated in the Oculus Rift VR head-mounted display is valid, it was compared to the yaw measured by sensors of the Optotrak 3020 computerised tracking system (Northern Digital, Waterloo, ON, Canada). Two active markers of the Optotrak system were placed symmetrically on top of the VR headset to measure the yaw.

Twenty rotations were made to produce a convenient number of samples to validate the Oculus Rift output. Normality of the range of motion measured by the Oculus Rift and the Optotrak system was assessed separately by visual inspection of their q-q plots and box plots. A Shapiro-Wilks test was carried out on the range of motion. The range of motion did not deviate from a normal distribution. Therefore, the Pearson correlation coefficient was used to determine the measure of linear dependence between the two groups. This resulted in a correlation coefficient of 1.00, p<0.001. When comparing the range of motion of the two groups, an average offset of 0.8 degrees ([0.7 degrees - 1.0 degrees] 95% confidence interval) was found between the VR headset and the Optotrak system. However, as this offset was constant across the different measurements, it was concluded that the accuracy of the output produced by the Oculus Rift VR headset was sufficient for the purposes of the current research project, because the outcomes assessed are the relative ranges of motion, not the absolute ranges of motion.

With previous versions of the Oculus Rift, drift appeared to be an issue because of approximately 6 degrees drift in the first 3 seconds of measurement [1]. Therefore, it was necessary to determine the drift in the current version. This was done by placing the Oculus Rift on a steady surface, and analysing the rotation that was measured by the VR headset during six measurements of 1 minute (in the current research project, participants were in an environment for no more than 20 seconds, after which the sensors of the Oculus were reset), also looking at the different gains (so 2 times 1 minute for each gain). Results throughout these six measurements were comparable, with minor fluctuations around the 0 degrees axis. The maximal deviation from 0 degrees measured towards both the left and the right was 0.04 degrees ([0.03 degrees - 0.05 degrees] 95% confidence interval) and the mean error during all measurements was 0.08 degrees ([0.07 degrees - 0.09 degrees] 95% confidence interval) when adding the errors of both directions. For the current research project, these minor errors posed no threat to the internal validity.

References

[1] Xu X, Chen KB, Lin JH, Radwin RG. The accuracy of the Oculus Rift virtual reality head-mounted display during cervical spine mobility measurement. J Biomech. 2015 Feb;48(4):721-4.
