# Supplementary material for: Using Visual Feedback Manipulation in Virtual Reality to Influence Pain‐Free Range of Motion in People with Nonspecific Neck Pain
Source: Pain Pract. 2020 Dec 20;21(4):428–37. doi: 10.1111/papr.12971 (PMC8048536; doi:10.1111/papr.12971)
Supplement: Supplementary file 2 — Appendix S2. A boxplot showing more detailed information regarding the effect of visual feedback manipulation on pain‐free range of motion. Figure S1. Effect of visual feedback manipulation on the pain‐free range of motion in people with subacute, chronic≤ 24 months, and chronic> 24 months neck pain. [file PAPR-21-428-s002.docx]

**Appendix S2:**

**FIGURE S1**


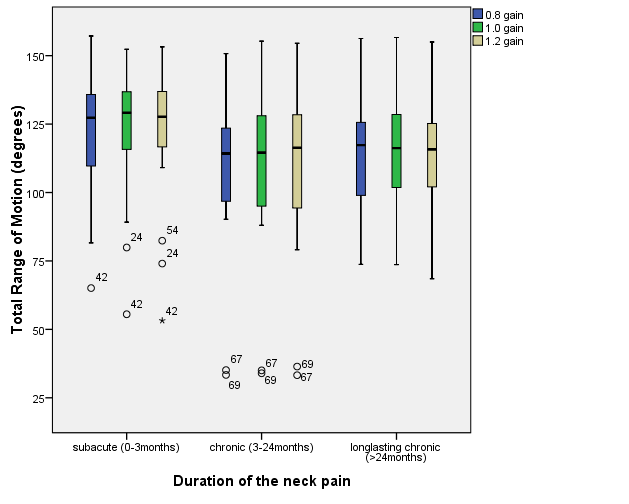


**Fig. S1**. Effect of visual feedback manipulation on pain-free range of motion in people with subacute, chronic_≤ 24MONTHS_, and chronic_> 24MONTHS_ neck pain
